# RETRACTION: Effect of Barbed versus Standard Sutures on Wound Complications in Total Knee Arthroplasty: A Meta‐analysis

**DOI:** 10.1111/iwj.70508

**Published:** 2025-04-18

## Abstract

**RETRACTION:** WangY. , XuH. , ZhaoY. , WangT. , ZhouH. , “Effect of Barbed versus Standard Sutures on Wound Complications in Total Knee Arthroplasty: A Meta‐analysis,” International Wound Journal 20, no. 10 (2023): 4130–4137, 10.1111/iwj.14307.37519132 PMC10681399

The above article, published online on 31 July 2023, in Wiley Online Library (http://onlinelibrary.wiley.com/), has been retracted by agreement between the journal Editor in Chief, Professor Keith Harding; and John Wiley & Sons Ltd. Following an investigation by the publisher, all parties have concluded that this article was accepted solely on the basis of a compromised peer review process. The editors have therefore decided to retract the article. The authors did not respond to our notice regarding the retraction.

**RETRACTION:** Y. Wang , H. Xu , Y. Zhao , T. Wang , H. Zhou , “Effect of Barbed versus Standard Sutures on Wound Complications in Total Knee Arthroplasty: A Meta‐analysis,” International Wound Journal 20, no. 10 (2023): 4130–4137, 10.1111/iwj.14307.37519132 PMC10681399

The above article, published online on 31 July 2023, in Wiley Online Library (http://onlinelibrary.wiley.com/), has been retracted by agreement between the journal Editor in Chief, Professor Keith Harding; and John Wiley & Sons Ltd. Following an investigation by the publisher, all parties have concluded that this article was accepted solely on the basis of a compromised peer review process. The editors have therefore decided to retract the article. The authors did not respond to our notice regarding the retraction.

